# Wisdom of crowds benefits perceptual decision making across difficulty levels

**DOI:** 10.1038/s41598-020-80500-0

**Published:** 2021-01-12

**Authors:** Tiasha Saha Roy, Satyaki Mazumder, Koel Das

**Affiliations:** Department of Mathematics and Statistics, Indian Institute of Science Education and Research Kolkata, Mohanpur, Nadia, 741246 India

**Keywords:** Cognitive neuroscience, Computational neuroscience, Visual system

## Abstract

Decades of research on collective decision making has claimed that aggregated judgment of multiple individuals is more accurate than expert individual judgement. A longstanding problem in this regard has been to determine how decisions of individuals can be combined to form intelligent group decisions. Our study consisted of a random target detection task in natural scenes, where human subjects (18 subjects, 7 female) detected the presence or absence of a random target as indicated by the cue word displayed prior to stimulus display. Concurrently the neural activities (EEG signals) were recorded. A separate behavioural experiment was performed by different subjects (20 subjects, 11 female) on the same set of images to categorize the tasks according to their difficulty levels. We demonstrate that the weighted average of individual decision confidence/neural decision variables produces significantly better performance than the frequently used majority pooling algorithm. Further, the classification error rates from individual judgement were found to increase with increasing task difficulty. This error could be significantly reduced upon combining the individual decisions using group aggregation rules. Using statistical tests, we show that combining all available participants is unnecessary to achieve minimum classification error rate. We also try to explore if group aggregation benefits depend on the correlation between the individual judgements of the group and our results seem to suggest that reduced inter-subject correlation can improve collective decision making for a fixed difficulty level.

## Introduction

Collective decision making is omnipresent across different biological species. Animals often need to make decisions in groups, for example, about which activities to perform^1^, when to perform^2–7^ and which direction to travel in^1^. A group could, in principle, reach a decision despotically, where only the dominant member decides^8–10^ or democratically, where a majority of the group members decides^1,8^. In^11^, the authors model the fitness consequences of these two complementary decision-making strategies and show that democratic decisions are way more beneficial in animals primarily because they tend to incorporate the individual decisions of a large section of the group members and hence lead to less extreme group decisions. Empirical evidence of democratic behaviours^1,5–8,12^ demonstrates that collective judgement exists across several species. Humans have also been found to essentially benefit from making decisions as a group^13–15^, a phenomenon popularly known as ‘the wisdom of crowds’^16,17^ and several crucial decisions in various fields including medical, judicial and political are often made after consulting with an expert-group. Collective decisions benefit not only from social interaction; even the accumulation of expert/non-expert judgements through advanced algorithms can lead to significant enhancement in decision accuracy^18–20^. In^20^, the authors compared different aggregation algorithms and group size for pooling behavioural data for both single location and visual search tasks using synthetic stimuli and demonstrated that although majority pooling is comparable to linear combination in single location tasks, majority voting performs poorly in comparison to average and weighted average for visual search tasks. Using eye tracking, they show that in visual search tasks, the benefits arising from the aggregation of individual judgements via linear combination are primarily due to the foveated nature of the human visual system and variations in gaze patterns of the participants. In the case of real world images, like medical images, satellite images or natural scenes, individual perceptual decisions are often limited by image properties like spatial uncertainty, target obstruction, and noise in imaging procedure. Individual brains might encode different features of the stimulus, and hence multi-brain pooling should benefit the decision making process via noise reduction. We sought to examine whether the pooling of decisions using either brain (EEG signals) or behaviour (confidence ratings) data in a target-detection task on natural scene images could similarly capture additional sensory information using a linear combination rule. Specifically, we explored whether a linear combination of individual decisions outperforms the commonly used majority pooling algorithm^21^ in detecting objects in indoor/outdoor natural scene images. It has been previously shown that performance benefit can be enhanced by increasing group size^22,23^. Here, we analyzed whether fusing information from fewer individuals than the maximum group size results in a comparable level of performance benefit. Additionally, we studied the effect of task difficulty with respect to the information gain due to group decision. Finally, we explore the role of inter-subject correlation in relation to collective decision making.

Studies exploring the ‘wisdom of crowds’ effect on perceptual decision making have, so far, not investigated the implications of optimum group size, task difficulty, and inter-subject correlations on group performance using neural signal analysis. The current study has tried to systematically address the above-mentioned issues, for which, to the best of our knowledge, there is no available evidence. We show that the overall group performance improves as a function of group size. A gradual decrease in individual confidence and an increase in classification error rate of the participants is observed with increasing task difficulty. The error is significantly reduced upon combining the individual decisions using the group aggregation rule. We demonstrate that it is possible to achieve similar performance benefits using smaller group size instead of using all members of the largest group, which can potentially lead to benefits in terms of cost and computation time. It is generally believed that high correlations in participant judgements could imply common sources of variability in perception, hence reducing pooling benefits^19^. Inter subject correlation(ISC) has been previously shown to be a potential marker of engagement^24,25^. ISC was also shown to be a predictor of memory in the context of online educational videos^26^. Here we explored the Group-ISC in the context of perceptual decision making under varying task difficulty. The group inter subject correlation decreased as group decision accuracy increased across difficulty levels and it can be argued that ISC can be indicative of group decision benefit.

Overall, our results demonstrate that although the relative advantages of combining neural activity across multiple brains might depend on the inter-subject correlation across difficulty levels, group decisions achieve accuracy rates that far surpass that of the average individual.

## Materials and methods

### Ethics statement

The study was carried out following institutional guidelines. All experimental protocols were approved by the ‘Institute Ethics Committee’ of Indian Institute of Science Education and Research Kolkata, India. Participants gave written informed consent in accordance with the Declaration of Helsinki.

### Stimuli and display

The data set consisted of 7.8 inch $\times$ 7.8 inch 8-bit indoor or outdoor natural scene images with 400 target-present images and 400 target-absent images. The participants were at a distance of 30 inch from the display. Images subtended a visual angle of 14.81 degree $\times$ 14.81 degree.

### Experiment

#### Experiment 1

**Figure 1:**
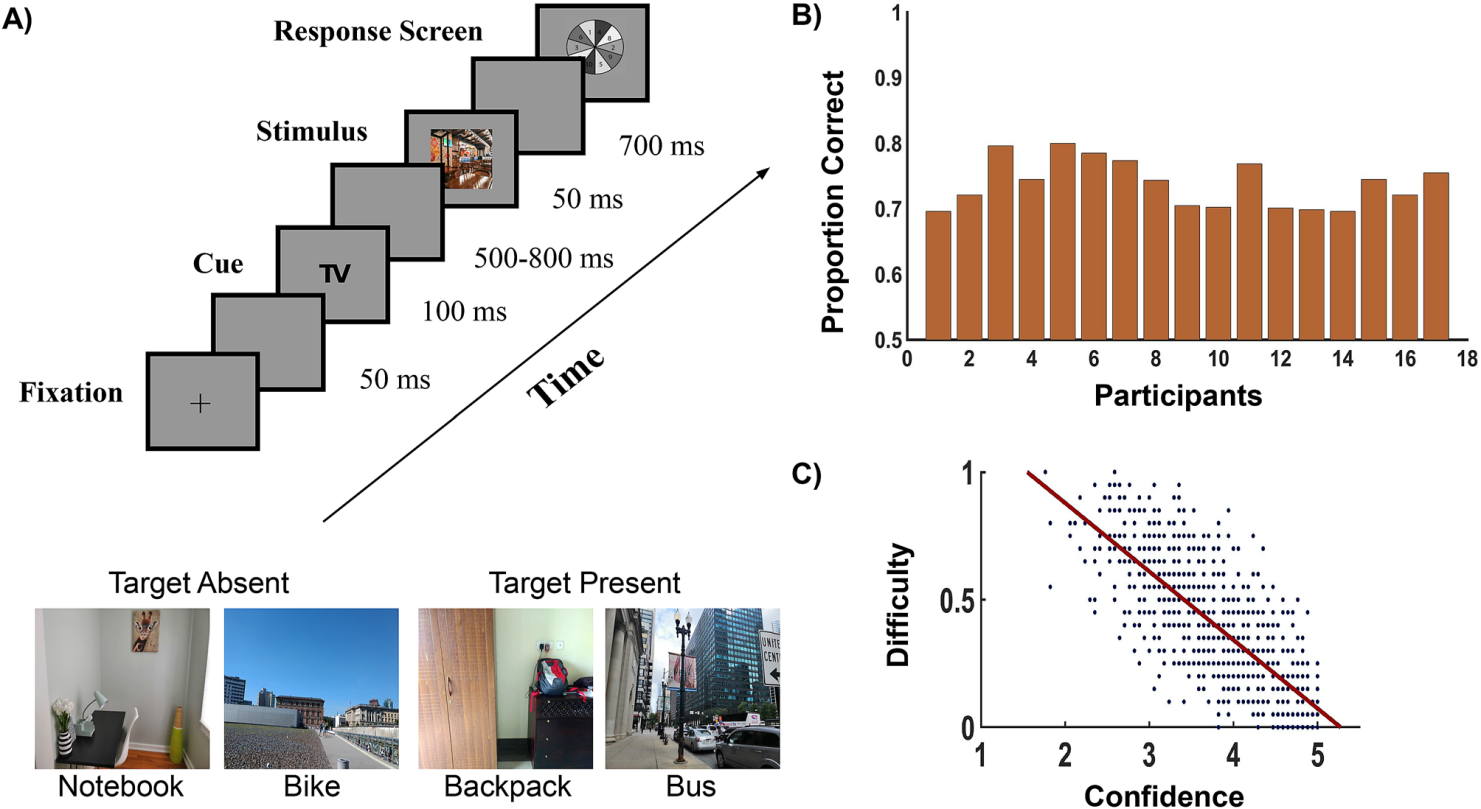
Experimental Protocol and Participant Behaviour. (**A**) Experimental Paradigm and example of Target Present and Target Absent natural scene images. (**B**) Behavioural performance of the seventeen participants. (**C**) Correlation between difficulty of the trials and mean individual confidence of the participants. Individual confidence decreases with task difficulty.

Eighteen healthy human volunteers (ages: 21–27, mean: 24.66, std: 2.42, seven female) participated in the study. All participants had normal or corrected-to-normal vision, disclosed no history of neurological problems, and were naive to the purpose of the study. The experiment consisted of 800 trials split into 32 successive sessions performed in one sitting. The total duration of the experiment was $\sim 1.5$ hours. All subjects were right handed and they used the same hand throughout the experiment to register their responses using the mouse click. One participant was not considered in the analysis due to a high degree of noise present in the neural data. The participants performed a visual search task where following the presentation of cues, they detected targets present in natural images. Targets were semantically consistent with the scene. Participants had no prior information about the target location or appearance. Participants fixated on a central cross and clicked anywhere on the screen when they were ready. After a delay of 50 ms, a target cue was presented for 100 ms followed by a variable delay of 500–800 ms. The stimulus was presented for 50 ms followed by a delay of 700 ms after which the response screen appeared. The participants reported their decision using a 10-point confidence scale (numbers 1 to 10 were displayed randomly to avoid any motor bias) with a rating of 1 indicating complete confidence that the target was present and a rating of 10 indicating that target was absent with complete confidence (see Fig. 1A).

#### Experiment 2

To assess the difficulty of the trials, another behavioural experiment was conducted with 20 new participants (ages: 23–28, mean: 24.5, std: 1.32, 11 female) with the same set of images as in Experiment 1 and the same set of target-cues. After each stimulus presentation, the participant was asked to respond whether the task of detecting the absence/presence of the target within the natural-scene image seemed ‘Easy’ or ‘Difficult’ to them. The response screen consisted of the words ‘Easy’ and ‘Difficult’ on either side of the screen in random order. Tasks were labelled based on the proportion of participants rating a particular target detection task difficult. The 800 images were divided into 5 groups for further analysis.

### Data acquisition and preprocessing

EEG activity was recorded using a 64 channel active shielded electrodes mounted in an EEG cap following the international 10/20 system at a sampling rate of 512 Hz. Trials were band-pass filtered (0.01–40 Hz.), epoched, and then referenced using average referencing. Trials with ocular artifacts (blinks and eye movements) were detected using bipolar electro-occulograms (EOG) with amplitude exceeding ± 100 mV or visual inspection and not included in the analysis. The high dimensional (number of electrodes $\times$ number of time-points) EEG data was reduced to an one dimensional neural decision variable using multivariate pattern classifier^27^. The preprocessed EEG signals were time-locked to stimulus onset and included a 200 ms pre-stimulus baseline and 700 ms post-stimulus interval.

### Dimension reduction

Since the neural data is high dimensional and suffers from small sample size problem^27^, a principal component analysis (PCA) based non-linear feature extraction technique—‘Classwise Principal Component Analysis’ (CPCA)^27^ has been used to reduce the dimensionality of the EEG signals and extract informative features. CPCA has been used successfully in previous studies to extract the multivariate pattern of neural signals^28–33^. The main goal of CPCA is to identify and discard non-informative subspace in data by applying principal component based analysis to each class. In this technique, PCA is applied locally to each class rather than on the whole data. Once the data is reduced locally using PCA, the reduced subspace then becomes low dimensional. The PCA subspace is further reduced to extract discriminatory features using a linear feature extraction technique like Linear Discriminant Analysis (LDA). The features retained in CPCA are the ones that carry discriminatory information between the two classes. The classification is then carried out in the residual space, in which small sample size conditions and the curse of dimensionality no longer hold. The method is described in Supplementary Information thoroughly.

### Integrating behavioural and neural decision variables across multiple brains

The behavioural confidence ratings and the neural decision variables were combined separately following the two rules described here to generate group behavior and neural decisions separately.

#### Majority pooling algorithm

While combining the behavioural and neural decision variables, a criterion was fixed per participant on the basis of which the final outcome of the group decision was determined. For participant *j*, let the criterion be $c_j$. For behavioural data, confidence ratings range between 1 and 10, both points inclusive. $c_j$ is the value midway between them, i.e., $c_j$ is taken to be 5.5, for each *j*. However, in the case of neural data, $c_j$, the neural decision criterion, was varied across all possible values of the training neural decision variables such that the true positive rate is maximized. Hence it differed from one participant to another (see^19^ for details). For each trial *i* the number of participants deciding on the presence of the target is quantified as

$$N_i = \sum _{j=1}^N I_{[0,\infty )}(c_j-R_{i,j}),$$

where $R_{i,j}$ is the neural decision variable on the *i*th trial for the $j{th}$ individual, *N* is the number of participants in the group and

$$I_{[0,\infty )}(x) = {\left\{ \begin{array}{ll} 1 &{} \text{ if } x \in [0,\infty ),\\ 0 &{} \text{ otherwise } . \end{array}\right. }$$

The outcome of the group decision $G_i$ is then defined as follows:

If *N* is odd,

$$G_i = {\left\{ \begin{array}{ll} 1 &{} \text{ if } N_i > (N-1)/2,\\ 2 &{} \text{ if } N_i \le (N-1)/2. \end{array}\right. }$$

If *N* is even,

$$G_i = {\left\{ \begin{array}{ll} 1 &{} \text{ if } N_i > N/2,\\ 2 &{} \text{ if } N_i < N/2,\\ r &{} \text{ if } N_i = N/2, \end{array}\right. }$$

where *r* is a random decision (about presence/absence of target) drawn from a discrete uniform distribution with support $\{1,2\}$. Here the outcome ‘1’ denotes presence of the target and ‘2’ denotes the absence.

#### Weighted average pooling algorithm

 A decision variable $D_i$ was obtained by taking the weighted average of the confidence ratings/ neural decision variables for every trial *i* for a group of participants (refer to^19^). Mathematically, $D_i$ is represented as

$$D_i = \varvec{w}'\varvec{R_i},$$

where $\varvec{R_i}$, the column vector, consists of the ratings or neural decision variables of the participants in a group, and the column vector $\varvec{w}$ contains the linear weights.

For each random group of *N* participants, the behavioural ratings/ neural decision variables corresponding to 90% of the trials were used for training and rest for testing the performance of the algorithm. This was performed 10 times. The weights $\varvec{w}$ were estimated from the training data by maximizing the Fisher’s criterion function as follows:

$$\varvec{w}= S_w^{-1} (\varvec{\mu _1} - \varvec{\mu _2}),$$

where $\varvec{\mu _1}$ and $\varvec{\mu _2}$ are the mean rating/neural decision variable for present trials and absent trials, respectively, for *N* participants, and

$$S_w = \sum _{\varvec{R_i} \in C_1} (\varvec{R_i}-\varvec{\mu _1})(\varvec{R_i}-\varvec{\mu _1})' + \sum _{\varvec{R_i} \in C_2} (\varvec{R_i}-\varvec{\mu _2})(\varvec{R_i}-\varvec{\mu _2})',$$

with $C_1$ and $C_2$ denoting the class of present trials and absent trials, respectively. But before inverting $S_w$, to tackle the issue of singularity (if any), it is regularized as

$$S_w(\gamma )= S_w + \gamma \varvec{I}_{N \times N},$$

where $\gamma$ is the ridge parameter. In our case, we chose $\gamma = 2$. $\varvec{I}_{N \times N}$ is the identity matrix, and *N* is the total number of participants in the group. The decision criterion *C*, which maximized the proportion correct, was also estimated from the training data. The group decision $G_i$, defined below, was obtained by comparing $D_i$ to criterion C. Define

$$G_i = {\left\{ \begin{array}{ll} 1 &{} \text{ if } D_i \le C,\\ 2 &{} \text{ if } D_i > C. \end{array}\right. }$$

The outcome ‘1’ denotes the presence of the target and ‘2’ denotes the absence.

We have limited the group decision rules to simple majority and weighted average pooling since these are the two most commonly used pooling algorithms^19,20^. Linear combination has been shown to be superior to majority voting on visual search tasks^20^ based on behavioural and eye tracking data. We have used both the pooling algorithms to fuse data from multiple brains using both behavioural and neural data.

### Computation of classification error rate

Quantifying the error in collective decision making becomes crucial towards understanding the performance benefits achieved by ‘the wisdom of crowds’. In the present study, the behavioural errors were calculated in a classification setup where the participants’ confidence ratings were taken as the explanatory variables or the features, and the ground truth (absent/present) of the trials was used as the binary categorical variable. Weights were calculated using 10-fold cross-validation^34^ on the trials as before. The absolute difference of the posterior probabilities obtained from classification and the ground truth was taken as the classification error rate.

To fully evaluate the potential of multi-brain computing for perceptual categorization, it is also important to quantify the error in predicting the ground truth using the neural information from multiple brains. The high-dimensional (59 electrodes $\times$ 354 time-points) EEG data coming from each participant was reduced to a single dimension using a multivariate feature extraction algorithm, discussed before in ‘Dimension Reduction’ subsection (^27^, see Supplementary Information for details). For each group, the weighted average decision rule was used to combine the neural information across the individual participants and the posterior probabilities of the classification were used to compute the errors.

In both cases, for every group size *N*, 100 distinct samples each containing *N* participants were generated, and the mean error across these 100 groups of size *N* was extracted per trial. Next, trials were categorized according to their difficulty levels. This allowed us to compare the errors across group sizes and task difficulty levels.

While it is well-known that collective judgement is more accurate than individual decisions, whether increasing the group size always results in a better group performance is still debatable. To address this, we performed pairwise comparisons between the classification error rates of groups of different sizes across all difficulty levels using the Wilcoxon rank sum test^35^. Benjamini-Hochberg method^36^ was used to correct for multiple hypothesis testing related to the different difficulty levels.

### Inter-subject correlation of a group

The Inter-subject correlation of a group (or the Group-ISC) is defined to be the average of the pairwise correlations of the members of that group. To find the relationship between group inter-subject correlation and task difficulty, we consider pairwise correlations of participants’ behaviour across trials of a particular difficulty level. For a group of size $N (>1)$ we take mean of the $$\left( {\begin{array}{c}N\\ 2\end{array}}\right)$$ pairwise correlations. For $N<17$, this procedure was repeated 100 times, generating distinct samples of *N* participants every time, and the Group-ISC was extracted (for $N=17$ only one distinct sample is possible so, the procedure is performed on one such sample).

Assuming that the neural signal acquired using EEG is reflective of the behavioural decision of the participant, group inter-subject correlation should be obtainable from the EEG data as well. Post-stimulus pre-processed EEG data was used to generate the inter-subject correlations using correlated component analysis^37^ based on the concept of finding the linear combinations of electrodes that have maximum correlations between participants. Here components are found using the following steps:

**Step 1: ** The cross-covariance of the electrodes in participant *k* with the electrodes in participant *l* is calculated as

$$R_{kl}= \frac{1}{T \times TR} \sum _{t=1}^{T} \sum _{tr=1}^{TR}(\varvec{x_k}(t,tr)-\overline{\varvec{x_k}})(\varvec{x_l}(t,tr)-\overline{\varvec{x_l}})',$$

where *T* and *TR* are the total number of time points and trials, respectively, vector $\varvec{x_k}(t,tr)$ represents the voltages recorded across all electrodes at time *t* for trial *tr* in participant *k*, and $\overline{\varvec{x_k}}$ is their mean value over all time points of all trials.

**Step 2: ** The pooled between subject cross-covariance is calculated as

$$R_b= \frac{1}{n(n-1)} \sum _{k=1}^{n} \sum _{l=1,l\ne k}^{n} R_{kl},$$

where *n* is the total number of participants.

**Step 3: **The pooled within-subject cross-covariance is calculated as

$$R_w= \frac{1}{n} \sum _{k=1}^{n} R_{kk}.$$

**Step 4: ** The largest eigenvalue of $R_w^{-1}R_b$ and the corresponding eigenvector have to be calculated. This vector captures the largest correlation between the participants. But before inverting $R_w$, to tackle the issue of singularity (if any), it is regularized as

$$R_w(\gamma )= R_w + \gamma \varvec{I}_{E \times E},$$

where $\gamma$ is the ridge parameter. In our case, we chose $\gamma = 0.005$. $\varvec{I}_{E \times E}$ is the identity matrix, and E is the total number of electrodes.

**Step 5: ** Let $\varvec{v}$ be the eigenvector corresponding to the largest eigenvalue of $R_w(\gamma )^{-1}R_b$. Projecting $(\varvec{x_k}(t,tr)-\overline{\varvec{x_k}})$ in the direction of $\varvec{v}$ we get a component

$$y_{k}(t,tr)= \varvec{v}' (\varvec{x_k}(t,tr)-\overline{\varvec{x_k}}),\, k \in \{1,2,\ldots ,n\}.$$

$y_{k}(t,tr)$ is a linear combination of all the voltages of electrodes (centered about their mean) with weights being determined by the components of the eigenvector $\varvec{v}$.

The pairwise correlation of participant *k* and *l* is given by

$$\rho _{kl} = \underset{1 \le tr \le TR}{\text{ median }}\left( \frac{\frac{1}{T}\sum \nolimits _{t=1}^{T}{y_k(t,tr)y_l(t,tr)}}{\sqrt{\sigma _{k}^2(tr)\sigma _{l}^2(tr)}}\right) ,$$

where $\sigma _{i}^2(tr) = \frac{1}{T}\sum \nolimits _{t=1}^{T} y_{i}^2(t,tr)$, for $i= k,\, l$, respectively. The Inter-subject correlation of a group is calculated taking the average of the pairwise correlations of its constituent members.

### Statistical tests

To compare the performance benefits arising out of pooling of individual decisions using the majority pooling algorithm and the weighted average algorithms, one-sided paired t-tests were conducted. Further a two-sample t-test was performed in a control study where we check whether the performance benefit achieved due to combining electrodes from multiple brains is significantly higher than the performance benefit achieved due to addition of electrodes from the same brain. Two-sample t-test, with Benjamini-Hochberg to control false discovery rate (FDR), was used in ERP analysis as well.

We used a two-way ANOVA to test for main effects of group size and difficulty level on the classification error rates of collective judgement. A three-way ANOVA was used to explore the main effects of group size, difficulty level and time on proportion corrects. The epoched EEG signals were segmented into 40 ms windows and classification was performed individually in these windows. To inspect main effects of group size and difficulty level on the Group-ISC, a two-way ANOVA was performed. After implementing ANOVA, “Tukey’s honestly significant difference procedure”^38^ was carried out to figure out the difficulty levels where the Group-ISCs were significantly different.

Wilcoxon rank sum test was used to test for equality of median classification error rates between different group sizes. The same non-parametric test was applied for comparing the median proportion correct between two difficulty levels at different time windows. Another nonparametric test, Wilcoxon signed rank test was implemented to compare the median of Group-ISC at difficulty level 1 with the median of Group-ISC at difficulty level 5 for different time windows. For each case, the issue of multiple hypothesis testing arises as the testings need to be done for either different group sizes or different time windows. To control FDR we used Benjamini–Hochberg method^36^ in each case.

The sample correlation coefficients (*r*) between the difficulty levels and individual confidence were calculated separately for 17 participants. The significance of the correlation coefficients were tested for each participant using one-sample t-test. Further to utilize all sample correlation coefficient together, we transformed each *r* to *z* using Fisher’s Z-transformation and tested the hypothesis regarding the correlation coefficient using *z*-test. The association between the weighted average of the behavioural ratings and the linear combination of the neural decision variables for each difficulty level were measured by calculating the sample correlation coefficient between the two. The partial correlation coefficient between the weighted average of the behavioural ratings and the linear combination of the neural decision variables was also calculated. Significance of theses correlation coefficients were tested using one sample *t*-test.

## Results

The individual behavioural performance of the seventeen participants on the visual search (in natural scenes) task is shown in Fig. 1B. Response time and accuracy varied with decision confidence (see figures S1 C-D of Supplementary Information). Using logistic regression, it could be shown that RT successfully predicts single trial accuracy and for every individual, the odds ratio of accuracy decreases with increasing RT (refer to figure S1 E of Supplementary Information).

### Association between mean confidence and task difficulty

We hypothesize that individual confidence of the participants on their decision should decrease as the perceptual task becomes difficult. In this experiment, the difficulty of the task was quantified by the proportion of participants from Experiment 2 who rated it difficult. Confidence ratings were converted to individual confidences and could take only integer values from the interval [1,5]. For example, a confidence rating of 1 or 10 would mean complete confidence that the target is present/absent in the image. In both cases, the confidence is taken as 5. A confidence rating of 5 or 6 would imply least confidence and would be assigned a confidence value of 1 in both cases. We calculated the correlation coefficients for each participant individually, and it is seen that the correlation coefficient turns out to be significantly negative for each one of them using one-sample t-test (max(*p* values)$<10^{-5}$). Further the null hypothesis that $\rho \ge 0$ against the alternative hypothesis $\rho <0$ is tested using the Fisher’s Z-transformation, where each $r_i$ (sample correlation coefficient for *i*th participant) is transformed to $z_i$ and test statistic is formed using $\overline{z}$. The z-value and the corresponding *p* value turned out to be − 52 and less than $10^{-100}$, respectively. For visual advantage, the scatter plot of the mean confidence value on the 800 trials across the 17 participants and the task difficulty is shown in Fig. 1C, with the overall correlation coefficient − 0.78 and the corresponding *p* value $< 10^{-100}$.

### ERP analysis

ERP analysis was performed on average referenced and baseline subtracted EEG signals for Target-Present and Target-Absent trials. Epochs of a particular channel were marked noisy if their respective absolute differences from the median exceeded 5 times the interquartile range. Such noisy epochs were not considered for further ERP analysis. After outlier detection, on an average 681 (± 78.82 std) trials remained for analysis.

**Figure 2:**
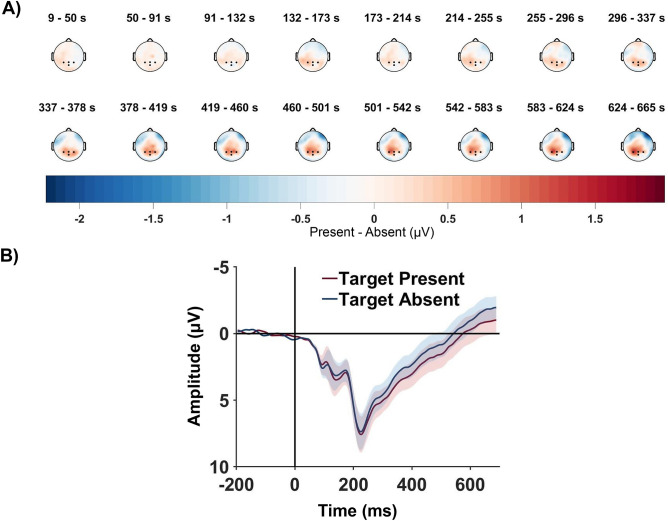
Univariate neural data analysis. (**A**) Topographic plot of Target Present—Target Absent difference wave. A dominant parietal activity is observed 300 ms onwards. The color-bar represents the magnitude of the difference wave in $\mu V$. (**B**) ERP waveforms corresponding to Target Present and Target Absent trials. Mean taken across 4 electrodes—P3, Pz, P4 and POz. Shaded area indicate ± SEM. The position of the 4 electrodes are marked using black dots in the topoplots of (**A**).

To elucidate whether the Target-Present and Target-Absent conditions induce different neural processing mechanisms, the grand average difference waveform was plotted (refer to Fig. 2A) on the scalp map using EEGLAB Toolbox^39^. A clear difference in Target-Present and Traget-Absent ERPs was visible across parietal electrodes 300 ms onwards post stimulus onset.

Additionally, statistical analysis was performed on the ERP signals to select electrodes having differences in activity for the Target-Present and Target-Absent conditions. Two sample t-test after multiple comparison ($p< 0.05$, FDR corrected in the window 220-350ms post stimulus onset) revealed significant differences in activity in the parietal electrodes. The ERP waveform in Fig. 2B is computed by taking the average across four electrodes : P3, Pz, P4 and POz. All four electrodes individually show significant differential activity post 250 ms. The ERP plot and the scalp map show the presence of the well known P300 component^40,41^ which is known to occur in target detection^42–44^. The P300 is elicited when observers respond to a stimulus-driven task and is independent of senosory modality^45^. It has also been known to contribute to attentional blink^46^.

### Performance benefits from integrating behavioural and neural decision variables across multiple brains

While the simple majority pooling algorithm has been effectively used in combining expert/non-expert judgements in single location tasks, the weighted average of individual decisions has been found to give superior benefits in simple visual search tasks where a small section of the group members often has more information relative to the majority. We exploit whether, in case of a more complicated scenario like visual search in natural scenes, the weighted average dominates the majority in terms of performance accuracy.

**Figure 3:**
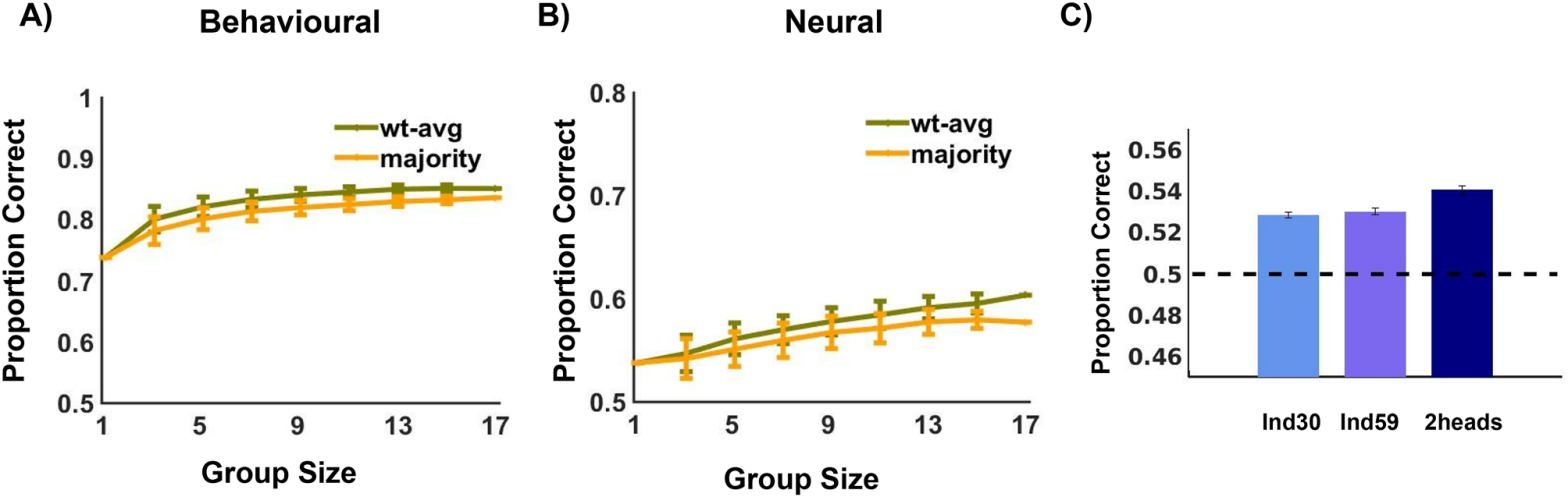
Group Aggregation. (**A**,**B**) Performance benefits achieved using the commonly used majority pooling algorithm and the weighted average algorithm across group sizes from behavioural (**A**) and neural (**B**) data. The proportion of correct trials is consistently more upon combining the individual decisions using the weighted average rule. Error bars indicate ± SD. (**C**) Control study to check that the the performance benefits achieved are not due to addition of more electrodes but due to multiple brains. The PC increases when doubling the number of heads relative to doubling the number of electrodes. ‘Ind30’ denotes single brain with 30 electrodes, ‘Ind59’ denotes single brain with all 59 electrodes and ‘2heads’ denotes double brain with 30 electrodes each. Error bars denote ± SEM.

To estimate the benefits of combining the behavioural and neural decision variables, 400 random groups were generated per group size, and the expected group decision was computed using the majority and weighted average pooling algorithms. The group performance was compared with the expected individual performance. Figure 3A-B show the mean performance accuracy in terms of Proportion Correct (PC) per group size, for behavioural and neural data, respectively. Clearly, with an increase in group size the PC increases for both the pooling algorithms. The maximum performance benefits achieved by group decision (i.e., by aggregating data from all 17 participants) is 15% for behavioural data and 12% from neural data using weighted average rule and 13% and 7%, respectively, using majority rule. On comparing the performance across the two algorithms per group size, we see notable superior performance of the weighted average rule ($p < 10^{-5}$ across all odd group sizes (from 3 to 15), FDR corrected, paired t-test, df = 399 ). This holds irrespective of whether we combine behavioural ratings of the group members or the neural decision variables.

**Figure 4:**
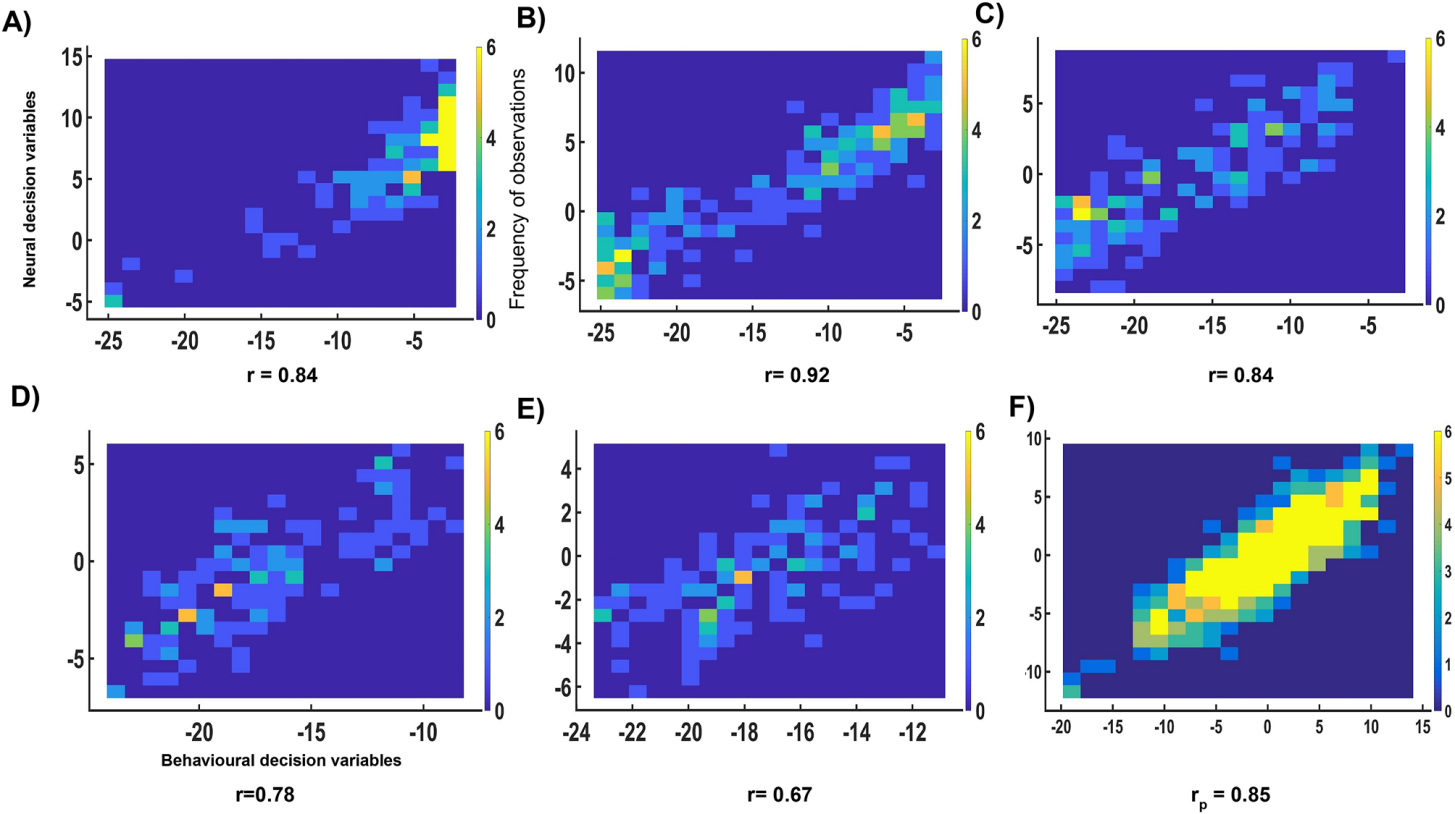
Relationship between Behavioural and Neural Decision Variables (**A**)–(**E**) Relationship between the behavioural and neural decision variables from the training data across difficulty levels 1 to 5. (**F**) Partial correlation between the behavioural and neural decision variables from the training data after controlling for effect of difficulty. Colour bars represent the frequency of observations.

**Figure 5:**
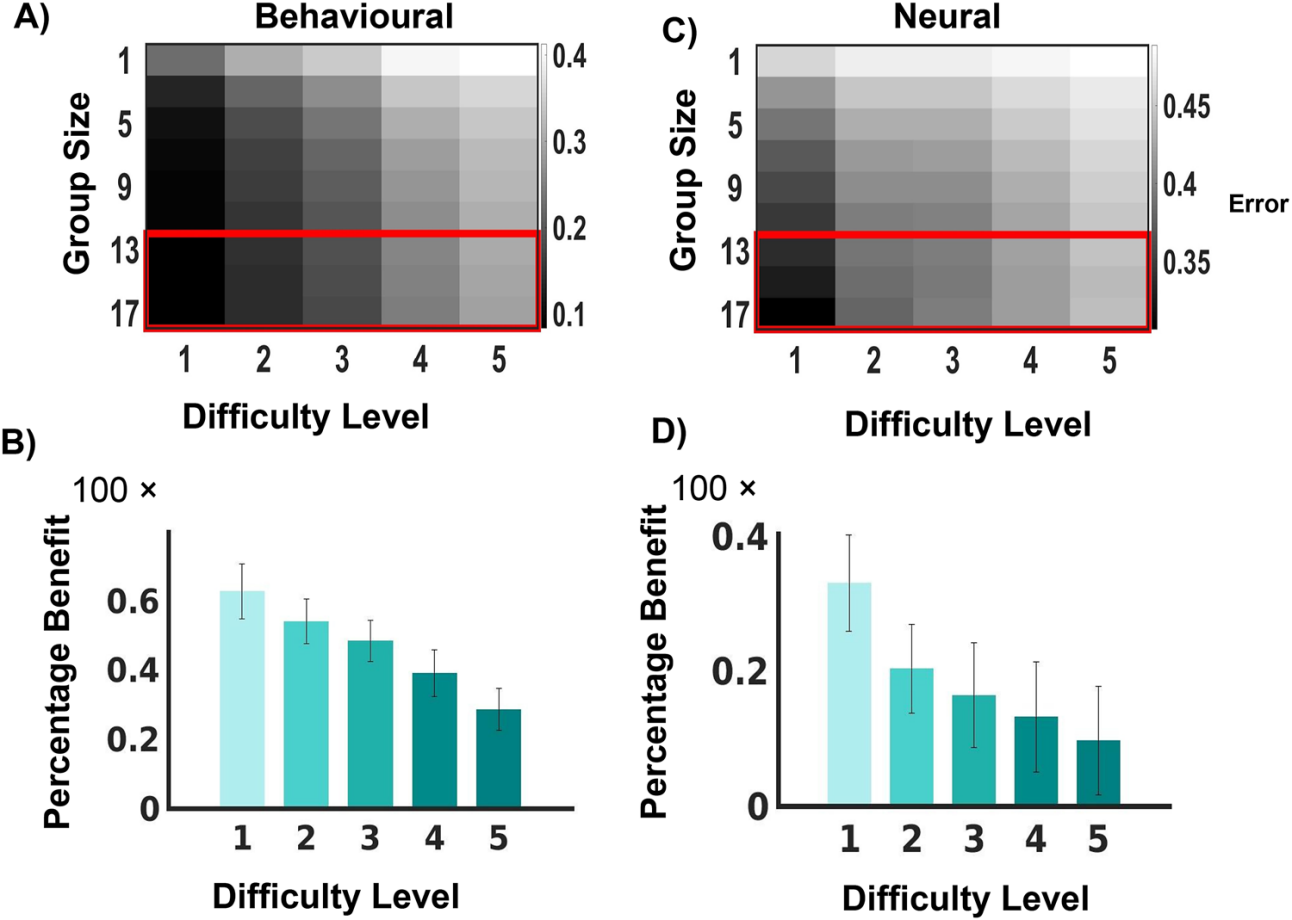
Group Aggregation and Task Difficulty. (**A**) and (**C**) depict the classification error rate across group sizes and difficulty levels for behavioural and neural data. Classification error rate increases with increasing task difficulty. This error is significantly reduced upon combining the individual decisions using the group aggregation rule. The colour bars indicate the magnitude of classification error rate. (**B**) and (**D**) show percentages of the benefit of group decision making (maximum group size vs individual) as a function of difficulty level for behavioural and neural data, respectively. Benefits reduce with increasing difficulty. Error bars represent ± SD.

**Figure 6:**
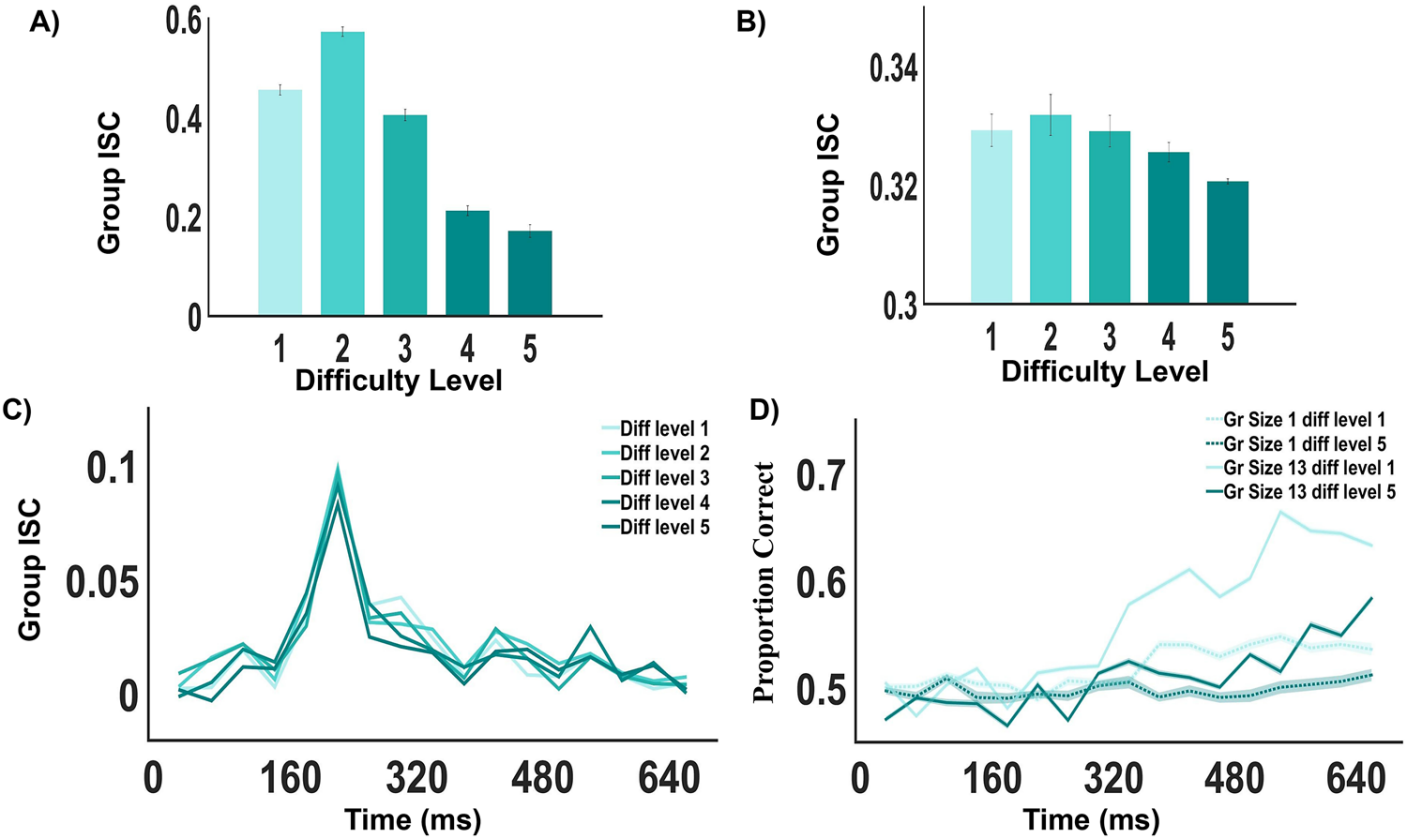
(**A**) and (**B**) Group-ISC as a function of task difficulty in behavioural and neural data, respectively. In both cases, Group-ISC is lower at difficult trials. Error bars denote ± SEM. (**C**) Neural timeline of Group-ISC at different difficulty levels. A sudden peak in the Group-ISC is noticed in the time window 200-240 ms after stimulus onset. The value is reduced with increasing task difficulty. In figures (**A**–**C**) the Group-ISC is maximum at the 2nd difficulty level and minimum at 5th difficulty level. (**D**) Neural activity in multiple brains across time at difficulty levels 1 and 5 for individual and group decision (Group Size 13).

While it is clear that inclusion of information from multiple brains is associated with an improved performance of the classifier, it needs to be verified that this is not solely due to an increase in the size of the data used for classification (regardless of whether the data comes from a single brain or multiple brains). If the classifier performance improved because of an increase in the volume of the data alone, then the performance of the classifier should not be very different when fusing neural signals from the two groups of electrodes from two different brains and when clubbing neural information from two groups of electrodes from single brains. We tested this by comparing performance using multiple brains and using a single brain but keeping the total number of electrodes comparable in both cases. The procedure was repeated 100 times by randomly sampling two brains and choosing two random groups of electrodes of size 30. We also showed the results using 30 electrodes from a single brain for comparison. But multivariate analysis reveals that combining information from multiple brains has a unique benefit on decision making (refer to Fig. 3C). Doubling the number of electrodes definitely leads to an information gain, but that due to doubling the number of heads clearly surpasses it (two-sample t-test, $t = 3.9204$, $p= 6 \times 10^{-5}$, df = 198).

### Correlation between groups’ behavioural and neural decision variables across difficulty levels

To explore the relationship between the collective stimulus-driven neural signal and the group decision confidence, we evaluate the correlation between the weighted average of the behavioural ratings and the combination of the neural decision variables. If the neural decision variable reflects the group decision regarding the presence/absence of the target in the natural scene, we expect a significant positive correlation between the weighted average of the participants’ confidence ratings and the weighted average of the neural decision variables from multiple-brains. Figure 4A-E depicts a scatter plot of the resulting behavioural and neural decision variables across all difficulty levels. The linear combination of behavioural confidence ratings and the integration of the neural decision variables of all seventeen brains are positively correlated across all difficulty levels ($r=0.84,0.92,0.84,0.77,0.67$, respectively, with increasing difficulty). All correlations are significant ($p< 10^{-15}$ for all five difficulty levels). Partial correlation between the neural and behavioural decision variables while controlling the effect of difficulty level was found to be 0.85 ($p < 10^{-50}$). Our results show that a significant correlation exists between behavioural and neural decision variables irrespective of difficulty levels. The corresponding scatter plot is provided in Fig. 4F.

### Classification error rate as a function of group size and difficulty level

While from Fig. 3A,B it is evident that aggregating individual decisions into a group decision increase the accuracy of the decision, a relevant question that arises is whether group size and difficulty level of the task plays any significant role in this net information gain using multiple individuals. Classification error rates of collective judgement were computed for each group size, and each difficulty level (average classification error rates are depicted in Fig. 5A,C, for behavioural and neural data, respectively), and a two-way ANOVA was performed. Significant main effects of both group size ($p< 10^{-20}$, $F = 7.96$, df= 6) and difficulty level ($p< 10^{-20}$, $F = 143.89$, df= 4) were observed. Similar effects were seen while considering the error in judgement from the neural group decision variables. Main effects of group size ($p< 0.005$, $F=3.55$, df= 6) and difficulty level ($p< 10^{-20}$, $F=13.73$, df = 4) were significant. The benefit of group decision making across the five difficulty levels were computed and a reduction in the percentage benefit in classification error rate was identified with increasing difficulty (Fig. 5B,D, for behavioural and neural data, respectively).

We try to figure out whether the information gain from group decision making is stable across all group sizes or is always more for larger groups. Pairwise comparisons between all pairs of groups, after correcting for multiple comparisons across the five difficulty levels, revealed that increasing the group size gave an advantage up to about two-thirds of our maximum group size (seventeen). In our case, there was not any significant difference between the classification error rates of thirteen or fifteen-member groups from seventeen-member groups across difficulty levels two to five (Wilcoxon rank sum test, $0.1<p <0.4$, FDR corrected) for both neural and behavioural performance. Hence we can conclude that optimum group size does not necessarily imply the maximum group size considering the improvement in performance.

### Integration of neural activity in multiple brains across time and difficulty levels

To assess whether the benefits of collective decision making arise in the early perceptual processing stage or late decision making stage, we evaluate classifier performance on the EEG data for different time windows. Epochs were divided into temporal windows spanning $\sim$ 40 ms each. Multivariate pattern classification was conducted separately for different time windows using all electrodes. An ANOVA was performed on these time-windowed PC values, with factors difficulty level, group size and time, revealed the significant main effect of all three factors on the classification accuracy ($p<10^{-20}$ in all three cases, $F = 5768.55, \, 479.8,\, 2003.77$ and df = 4, 6, 16, respectively). Figure 6D shows the neural timeline for individual versus group decision (for group size 13) at difficulty levels 1 and 5, respectively. Wilcoxon rank sum test was performed to compare the proportion correct between the two difficulty levels for a fixed group size and between the two group sizes for a fixed difficulty level, respectively. PC at the first difficulty level was consistently higher than PC at the highest difficulty level (right tailed test, $p<10^{-5}$, FDR corrected) for individual decision (post 360 ms) as well as group decision (the group of size 13, post 320 ms). For the first difficulty level post 280 ms, a considerable benefit was achieved using group decision ($p<10^{-4}$, FDR corrected, for group size 13, Group PC > Individual PC). But for the fifth difficulty level, i.e., for tasks of maximum difficulty, the consistent significant difference between individual and group decision (Group PC > Individual PC) was observed much later (after 560 ms, $p<10^{-5}$, FDR corrected). It appears that benefits accrued due to group decisions in visual search are dependent on task difficulty; greater the difficulty level later the neural benefit.

### Relationship between group inter-subject correlation and task difficulty

The Group Inter-Subject Correlation (Group-ISC) is computed by taking the mean of the pairwise correlations of the group members. Pairwise correlations between the confidence ratings of the participants on trials of the same difficulty are extracted to find the behavioural Group-ISC. An ANOVA was performed on these Group-ISCs, with factors difficulty level and group-size, revealed the significant main effect of difficulty level on the Group-ISC ($p<10^{-50}$, $F = 17091.57$, df = 4) as well as a significant main effect of group-size ($p<0.01$, $F = 2.99$, df = 6). Multiple comparison tests were performed using ‘Tukeys honestly significant difference procedure’^38^ to find out in which difficulty levels the Group-ISCs were significantly different. All pairs of difficulty levels showed a significant difference ($p<0.0001$ in all cases) in their mean Group-ISC values. Figure 6A shows the mean Group-ISC across difficulty levels. The Group-ISC decreases after the 2nd difficulty level, depicting that with the increase in difficulty, the correlation between individual decisions of the participants reduces.

To see whether this randomness in the behaviour of the participants with increasing task difficulty is also reflected in their neural activity, we found the Group-ISC from the EEG data (see Material and Methods section for details). An ANOVA was performed on the Group-ISCs, with factors difficulty level and group-size, revealed the significant main effect of difficulty level on the Group-ISC ($p< 10^{-20}$, $F = 29.44$, df = 4) as well as a significant main effect of group-size ($p<10^{-20}$, $F =38.19$, df = 6). Multiple comparison tests revealed no significant difference between the mean Group-ISCs of difficulty level 1 and 2 ($p= 0.1495$), 1 and 3 ($p= 0.9999$) and 2 and 3 ($p=0.1063$). All other pairs of difficulty levels showed a significant difference ($p<0.01$ in all cases) in their mean Group-ISC values. Figure 6B shows that the mean neural Group-ISC decreases after the 2nd difficulty level, similar to its behavioural counterpart (Fig. 6A).

To explore how the Group-ISC evolves over time, the post-stimulus neural data was divided into distinct 40 ms time windows as before. Group-ISCs of the participants were computed separately in these time windows. The values increase after 160 ms of stimulus onset and reaches its peak in the 200–240 ms time window (refer to Fig. 6C). A sharp decline is noticed thereafter across all difficulty levels. The Group-ISC is highest for difficulty level 2 and lowest for difficulty level 5 at the peak. Wilcoxon signed rank test reveals that Group-ISC at difficulty level 1 is significantly greater than the Group-ISC at difficulty level 5 in the time window 200–440 ms post stimulus onset ($p < 0.05$, FDR corrected). However, irrespective of tasks of varying difficulty, the highest average correlations between group members are observed in the same time frame, and the task difficulty determines the magnitude of this effect.

All data analyses were performed using MATLAB 2018.

## Discussion

Enhanced decision making accuracy achieved via collective judgement has been reported in the case of several animals—from insects to humans. In the case of perceptual decisions, although all individuals from the same species share a common neural architecture, the sensory processes of the different individuals might encode different information about the task. Integration of the neural activity across a group of participants will incorporate the collective information from multiple brains and reduce the effects of neural noise, leading to a more holistic representation of the information about the task and a consequent improvement in decision accuracy. In the current study, we demonstrate that using multi-brain computing, a neural pattern-based classifier can achieve higher accuracy at a visual search task using natural images than utilizing the EEG data from a single brain. Another separate analysis revealed that, when keeping the data size constant (i.e., fixing the total number of electrodes), pooling neural information across multiple brains accomplishes unique advantages to the classifier performance. An important aspect of collective decision making is an aggregation of the independent contributions of the individuals into a collective output. The majority decision rule has arguably been the most common type of group aggregation algorithm used across species. A previous study^19^ using a face/car task compares three different aggregation rules and reports that benefits arising due to pooling of task-relevant neural activity across brains using weighted average gives the best performance accuracy closely followed by majority voting. Another study^20^ showed that the pooling benefits of the weighted average rule are significantly greater for visual search tasks than for single-location visual perceptual tasks and that the accuracy benefits of majority voting are less for visual search relative to averaging and weighted averaging of participants’ confidences. In the current study Electroencephalography (EEG) signals were recorded while participants performed a visual search task of detecting random targets in real scenes. Our results indicate that the presence or absence of targets can be predicted in natural scenes using multi-brain computing, and the weighted average of individual confidence ratings gives significantly more accurate judgement than the majority voting pooling algorithm. Accuracy gradually increases as the number of participants in a group increases as reported previously in^19,20^.

The visual stimuli, including indoor or outdoor natural scenes, varied in terms of content, clutter, contrast, or luminosity, making the target detection task more difficult in some cases than the rest. The effect of task difficulty on visual perception has been so far explored only at the individual level^47–52^ and not in case of group decisions. While it becomes clear that aggregating information from multiple brains improves the decision making accuracy, it is necessary to assess whether the information gain is uniform across stimuli of varying difficulty or not. Intuitively, an increase in task difficulty would imply a decrease in confidence on one’s own decision. Our analysis confirmed the same, with the mean individual confidence of the participants being negatively correlated with the difficulty of the trials. In fact, the classification error rate of the decision making task was inversely related to the task difficulty, as revealed from our single-brain decoding analysis. The error could be significantly reduced upon combining the behavioural/neural decision variables from multiple brains using the weighted average rule. The reaction time (Figure S1 B of Supplementary Information) was greater for incorrect trials. Reaction time decreased with increase in confidence level and was inversely proportional to behavioural accuracy, consistent with previous literature^53^. Although previous research using multi-brain data discussed group size effect^19,20^, almost all studies use the maximum available group size for reporting the maximum benefit in collective decision. Here, using statistical tests, we show that aggregating the behavioural/neural data from all available participants is unnecessary to achieve minimum classification error rate. Once an optimum group size is reached which produces significant gain in terms of performance benefit, increasing the group size results in negligible incre›ase in performance and only adds to the computational cost and resource allocation. In the current study, a group size of about two-thi›rd of our maximum group size was optimal for achieving the desired accuracy.

Decision making is typically believed to be arising in the later stages of sensory information processing. To explore whether the performance benefits of collective judgement in visual search tasks appear due to the integration of sensory information at decision stages we conducted a temporal window wise analysis of the EEG data coming from multiple brains across the different difficulty levels. If benefit arises at the later perceptual stage, we would expect that the advantages of integrating neural activity across individuals would be enhanced at the decision stage than at the early sensory stage. In fact, our results support this hypothesis. We showed that benefits in the later stage are higher than those in early sensory stages. Multivariate analysis across the post-stimulus time epochs also revealed that combining information from a group of optimal size, using the weighted average rule, increased decision accuracy. It was possible to attain a specific accuracy (achieved by an individual) much faster by pooling the neural information of multiple brains. But this effect diminishes with increasing task difficulty. However, collective performance still outperforms individual performance even for highest difficulty level thus showing the efficacy of multi-brain fusion.

ISC has been previously studied in the context of engagement, memory, and development^24,26,54^, but to the best of our knowledge, its role in perceptual decision making remains unexplored. We analyzed the group neural performance and Group-ISC over neural time scale and observe that the maximum group correlation occurred at 200–240 ms after stimulus onset for all difficulty levels. However, as seen from time window analysis, the increase in neural performance becomes prominent post 300 ms after inter-subject correlation reaches its peak. The finding is also consistent with the well known P300 component seen in our ERP analysis. It is interesting to note that the Group-ISC peak precedes the decision making stage post 300 ms. The variability between observers in encoding task related information can be evident in early sensory processing stage or at a later decision making stage. The Group-ISC timescale seems to suggest that there might be less variability between observers in the sensory stage while variability increases in the later decision stage. Integrating the decorrelated neural activity across observers during collective decision, the various sources of information present in different brains are considered thus boosting performance in the decision stage. Hence we propose that the Group-ISC can be an indicator of efficacy of benefit in the neural time-scale.

Multi-brain fusion finds its use in the field of neuroergonomics in the form of collaborative Brain Computer Interfaces (BCI)^55–57^. The use of optimum group size can be useful for designing real-time collabortive BCI applications as it would result in huge savings both in terms of resources and computation cost. We hope our results, especially use of optimum group size and relationship of performance gain with Group-ISC would help in designing better collaborative BCIs in future.

## Supplementary information

Supplementary material 1

## Data Availability

Analysis codes and related resources are publicly available here: (10.6084/m9.figshare.13276778). Further details and requests for additional resources should be directed to Koel Das (koel.das@iiserkol.ac.in).
