# Supplementary material for: Wisdom of crowds benefits perceptual decision making across difficulty levels
Source: Sci Rep. 2021 Jan 12;11:538. doi: 10.1038/s41598-020-80500-0 (PMC7804123; doi:10.1038/s41598-020-80500-0)
Supplement: Supplementary file 1 — Supplementary material 1 [file 41598_2020_80500_MOESM1_ESM.pdf]

Wisdom of crowds benefits perceptual decision making across difficulty levels

Tiasha Saha Roy[a], Satyaki Mazumder[a], and Koel Das*[a]

[a]Department of Mathematics and Statistics, Indian Institute of Science Education and Research Kolkata, Mohanpur, Nadia-741246, India

*Corresponding Author: koel.das@iiserkol.ac.in

Supplementary Information

Reaction Time Analysis

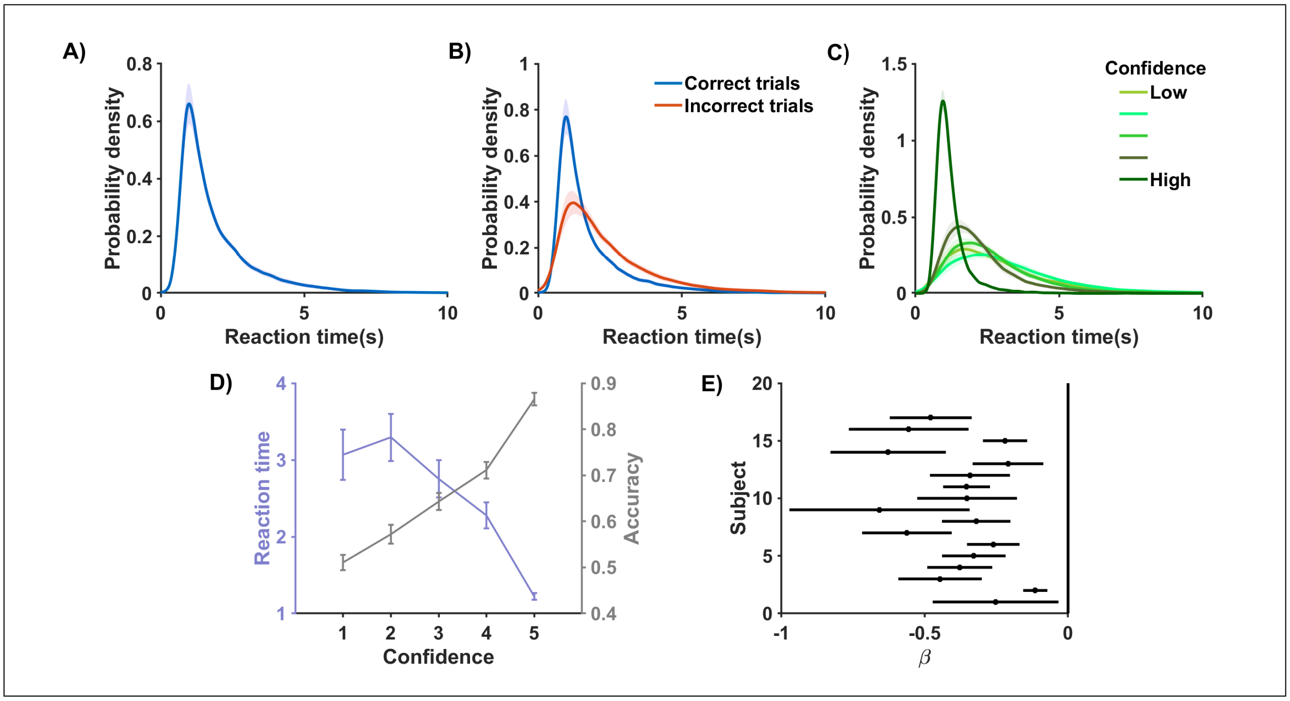

Figure S1: Reaction time and accuracy (**A**) RT distributions from stimulus offset, shown for all trials. (**B**) RT distributions shown for correct and incorrect trials separately. (**C**) RT distributions shown for trials of different confidence levels (1-5). In (**A-C**) the plotted RT distribution is the average taken over individual participant RTs. Probability distributions have been fitted to the individual RT data using MATLAB's 'fitdist' function. (**D**) Accuracy and mean RT as a function of confidence (1-5). (**E**) 95 % confidence intervals of logistic regression slopes (β), using RT to predict single-trial accuracy at individual level. Clearly the slopes of the each logistic regression are significantly less than 0.

Classwise Principal Component Analysis (CPCA)

Single-trial classification of EEG data is a challenging problem due to the high dimension low sample size problem. Under the small sample size conditions, a large portion of the data space is sparse and carries almost no useful information. Generally the non-informative subspace is discarded through extraction of a small set of useful features (feature extraction). In this study we have used a recently proposed non-linear computationally efficient, locally adaptable, pattern classification technique for tackling the HDLSS problem of EEG data. In this algorithm, the strength of PCA as a dimensionality reduction technique is exploited, while preserving the class-specific information to facilitate further classification. The main

idea behind the technique is to identify and discard an irrelevant subspace in data by applying PCA to each class. The classification is then carried out in the lower dimensional residual space.

Without loss of generlaity , let us assume it is a binary classification problem.

- Step 1 (Feature Extraction) : In this step a piecewise linear subspace is extracted from the training trials.

  Let $w_i (i = 1, 2)$ denote two classes with means μ_i, and covariances Σ_i. Let $x^* \in \mathbb{R}^n$ be the unknown test data. To represent x^* in the two subspaces S_1 and S_2 we use the following transformation,

$$x_i{}^* = F_i{}^T (x^* - \mu_i), \text{i = 1,2} \tag{1}$$

  where F_i is a matrix of order $n \times m_i{}'$ with the columns of F_i as the basis vectors of S_i. The two classes are transformed in a similar way. In the simplest case, F_i can be taken as $V_i \in \mathbb{R}^{n \times m_i}$, where V_i consists of the m_i (m_i' to be chosen) principal components of the class w_i. To ensure that class differences arising from the two means are accounted for, F_i is taken as

$$F_i = [V_i | V_b]$$

  where $V_b \propto (\mu_1 - \mu_2)$. To make all projections orthonormal, we orthonormalize the columns of F_i via the Gram- Schmidt process.

  There is a scope of betterment in term of classification accuracy, if simple feature extraction techniques are applied to the subspace S_i directly. If linear feature extraction techniques are used (e.g. LDA) with $T_i \in \mathbb{R}^{m_i' \times m}$, as the feature extraction matrix, then F_i can be taken simply as $[V_i | V_b] T_i$ and the rest mathematical formulation remains the same.

- Step 2 (Classification) : In this step unknown test trial is classified in one of the subspaces estimated during the feature extraction step. To complete the feature extraction process, one of the two subspaces has to be removed. This problem can be solved within a classification framework. The feature extraction process will then be a bi-product of the classification process. We elaborate it below. For simplicity, the classes are taken to be Gaussian with prior probabilities $P(w_i)$. Applying the Bayes classifier at the first subspace implies

$$P(w_{i1} | x_1{}^*) = \frac{P(x_1{}^* | w_{i1}) P(w_i)}{P(x_1{}^*)}, \text{ for } i = 1, 2, \tag{2}$$

  where

$$P(x_1{}^*) = \sum_{i=1}^{2} P(x_1{}^* | w_{i1}) P(w_i)$$

is the marginal probability of x_i^* and $P(w_{i1}|x_i{}^*)$, $i = 1, 2$, are the posterior probabilities of the two classes in the first subspace. Note that the conditional distribution x_1^* given w_{i1} remain Gaussian due to the linear transformation of x^* (see equation (1)), for $i = 1, 2$. In particular,

$$P(*|w_{i1}) \sim N({F_1}^T(\mu_i - \mu_1), {F_1}^T \Sigma_i F_1), \text{ for } i = 1, 2.$$

In a very similar manner, x^* is projected in the second subspace. The posterior probabilities of the two classes in the second subspace are then given by

$$P(w_{i2}|{x_2}^*) = \frac{P({x_2}^*|w_{i2})P(w_i)}{P({x_2}^*)}, \text{ for } i = 1, 2, \tag{3}$$

where

$$P({x_2}^*) = \sum_{i=1}^{2} P({x_2}^*|w_{i2})P(w_i)$$

and

$$P(*|w_{i2}) \sim N({F_2}^T(\mu_i - \mu_2), {F_2}^T \Sigma_i F_2), \text{ for } i = 1, 2.$$

Based on these posteriors we associate x^* to one of the two classes. After projecting x^* in the first class it is associated to class w_k with the maximum posterior $k = \arg\max_{i=1,2} P(w_{i1}|x_1^*)$, similarly x^* is assigned to class w_ℓ with the maximum posterior $\ell = \arg\max_{i=1,2} P(w_{i2}|x_2^*)$. Finally x^* is associated to class w_g by comparing the highest posterior probabilities obtained in individual subspaces, i.e., $g = \arg\max_{k,\ell} [P(w_{k1}|x_1^*) \quad P(w_{\ell 2}|x_2^*)]$.

Discriminative Filter Weights Although the features appearing in CPCA are abstract, the corresponding filters have a physical interpretation. By filters we mean the columns of F_i. The filter coefficients, when arranged into a spatiotemporal array, indicate brain areas and time scales which are engaged in encoding the differences between the two classes (target present/absent).
